# ‘Swollen heart’ in the course of acute intermittent porphyria associated with severe hyponatraemia

**DOI:** 10.1093/ehjci/jeac186

**Published:** 2022-09-07

**Authors:** Krzysztof Jaworski, Robert Wasilewski, Hanna Szwed, Jerzy Windyga, Rafal Dabrowski

**Affiliations:** Department of Coronary Artery Disease and Cardiac Rehabilitation, National Institute of Cardiology, Alpejska 42, 04-628 Warsaw, Poland; Department of Hemostasis Disorders and Internal Medicine, Institute of Hematology and Transfusion Medicine, Indiry Gandhi 14, 02-776 Warsaw, Poland; Department of Coronary Artery Disease and Cardiac Rehabilitation, National Institute of Cardiology, Alpejska 42, 04-628 Warsaw, Poland; Department of Hemostasis Disorders and Internal Medicine, Institute of Hematology and Transfusion Medicine, Indiry Gandhi 14, 02-776 Warsaw, Poland; Department of Coronary Artery Disease and Cardiac Rehabilitation, National Institute of Cardiology, Alpejska 42, 04-628 Warsaw, Poland

**Figure:**
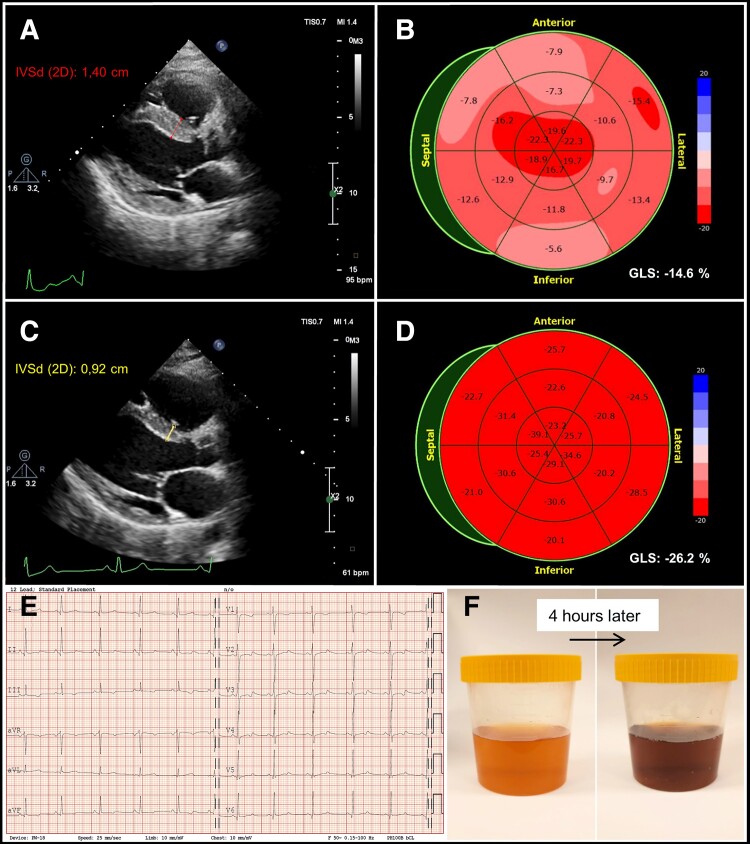


A 30-year-old woman was admitted to hospital due to abdominal pain, weakness of muscles, tachycardia, and elevated blood pressure (160/97 mmHg). Laboratory tests revealed severe hyponatraemia (107 mmol/L, normal 135–145). Based on the clinical picture, family history and increased excretion of porphobilinogen, acute intermittent porphyria was diagnosed. The following day, the patient reported chest discomfort. Electrocardiogram showed sinus rhythm of 95 bpm, no relevant ST-T changes. Troponin T, N-terminal pro-B-type natriuretic peptide, and norepinephrine concentrations were significantly elevated [30 ng/L (normal <14), 1390 pg/mL (<125), 577 pg/mL (<110), respectively]. Echocardiography demonstrated increased thickness of left ventricular walls (*Panel A*). No regional wall motion abnormalities were detected in 2D imaging although global longitudinal strain was decreased (*Panel B*). The patient was treated with haeme arginate, hypertonic saline, metoprolol, and her condition improved in 3 days. Control TTE showed complete resolution of all pathological findings (*Panels C–D*). Cardiac magnetic resonance revealed no structural heart disease. However, biphasic T waves and prolonged QTc interval were observed transiently in ECG (*Panel E*). In 1-year follow-up, the patient remained symptomless.

Acute intermittent porphyria is a genetically determined disorder of haeme biosynthesis. The main symptoms include abdominal pain, paralysis, and coma. Hyponatraemia occurs in some patients as a result of inappropriate antidiuretic hormone secretion. A bedside sign may be the darkening of urine after exposure to light (*Panel F*). In the presented patient, the mechanism of cardiac injury is uncertain and may have been related to severe hyponatraemia, catecholamine surge, or the toxicity of haeme precursors.

## Data Availability

The data underlying this article will be shared on request to the corresponding author.

